# Comparison of the Protective Effects of Ginsenosides Rb1 and Rg1 on Improving Cognitive Deficits in SAMP8 Mice Based on Anti-Neuroinflammation Mechanism

**DOI:** 10.3389/fphar.2020.00834

**Published:** 2020-06-10

**Authors:** Yujie Yang, Shanshan Li, Hong Huang, Jingwei Lv, Shanguang Chen, Alberto Carlos Pires Dias, Yujiao Li, Xinmin Liu, Qiong Wang

**Affiliations:** ^1^Affiliated TCM Hospital, School of Pharmacy, Sino-Portugal TCM International Cooperation Center, Southwest Medical University, Luzhou, China; ^2^Research Center for Pharmacology & Toxicology, Institute of Medicinal Plant Development (IMPLAD), Chinese Academy of Medical Sciences and Peking Union Medical College, Beijing, China; ^3^Institute of Food Science and Technology, Chinese Academy of Agricultural Sciences (CAAS), Beijing, China; ^4^Centre of Molecular and Environmental Biology (CBMA), SINO-PT Research Center, Department of Biology, University of Minho, Braga, Portugal; ^5^National Key Laboratory of Human Factors Engineering, China Astronaut Research and Training Center, Beijing, China

**Keywords:** ginsenoside Rb1, ginsenoside Rg1, Alzheimer's disease, SAMP8 mice, neuroinflammation

## Abstract

This present study was designed to investigate the different effects of ginsenosides Rb1 and Rg1 on improving cognitive deficits in 4-month-old SAMP8 mice. Mice were divided into six groups, including the SAMP8 group, the SAMP8 + Donepezil (1.6 mg/kg) group, the SAMP8 + Rb1 (30 and 60 µmol/kg), and SAMP8 + Rg1 (30 and 60 µmol/kg) groups. SAMR1 mice of the same age were used as the control group. Ginsenosides and donepezil were administrated orally to animals for 8 weeks, then the learning and memory ability of mice were measured by using Morris water maze (MWM) test, object recognition test and passive avoidance experiments. The possible mechanisms were studied including the anti-glial inflammation of Rb1 and Rg1 using HE staining, immunohistochemistry and western blot experiments. Results revealed that Rb1 and Rg1 treatment significantly improved the discrimination index of SAMP8 mice in the object recognition test. Rb1 (60 µmol/kg) and Rg1 (30, 60 µmol/kg) could significantly shorten the escape latency in the acquisition test of the MWM test in SAMP8 mice. Furthermore, Rb1 and Rg1 treatments effectively reduced the number of errors in the passive avoidance task in SAMP8 mice. Western blot experiments revealed that Rb1 showed higher effect than Rg1 in decreasing protein expression levels of ASC, caspase-1 and Aβ in the hippocampus of SAMP8 mice, while Rg1 was more effective than Rb1 in decreasing the protein levels of iNOS. In addition, although Rb1 and Rg1 treatments showed significant protective effects in repairing neuronal cells loss and inhibiting the activation of astrocyte and microglia in hippocampus of SAMP8 mice, Rb1 was more effective than Rg1. These results suggest that Rb1 and Rg1 could improve the cognitive impairment in SAMP8 mice, and they have different mechanisms for the treatment of Alzheimer's disease.

## Introduction

Alzheimer's disease (AD) is a neurodegenerative disease with complex pathogenic factors. Its main symptoms are cognitive impairment, execution disorder, memory impairment, obvious mental disorder, sleep disorder, and even behavioral abnormalities (Yang et al., 2017; Yutaro et al., 2018). Many studies have indicated the cardinal features of Alzheimer pathology are amyloid plaques, neurofibrillary tangles (NFTs), associated with astrogliosis and microglial activation (Lane et al., 2018; Boumenir et al., 2019). In recent years, increasing evidence suggests that neuroinflammation is a causal role in the pathogenesis of AD (Heneka et al., 2015; Zhang et al., 2015). Apoptotic process closely associated with inflammasome, and lead to neuronal cell death in AD (Xingxing and Tao, 2018). Microglia and astrocyte are the main cells involved in neuroinflammatory reactions. Activated microglia and astrocyte can produce pro-inflammatory cytokines such as tumor necrosis factor-α (TNF-α), interleukin-1β (IL-1β), and interleukin-6 (IL-6) (Kima et al., 2019).

Ginsenosides are the main active components of Panax ginseng C. A. Meyer, a traditional chinese herbal medicine, which has a wide range of pharmacological effects, such as anti-aging, anti-tumor, and nervous system protection, etc. In recent years, researches have indicated that ginsenosides play a pronounced positive role in the treatment of AD (Razgonova et al., 2019) and have memory enhancement effects (Wang et al., 2016). Ginsenosides Rg1 and Rb1 are the major ginsenosides in ginseng with identified neuroprotective effects. Rg1 can decreased Aβ level, attenuated hippocampal histopathological abnormalities and improved learning and memory in a rat model of AD induced by injection of soluble beta-amyloid peptide 1–42 (Ab_1–42_) into the hippocampus (Quan et al., 2013). Rb1 reversed memory impairment induced by aluminum (Al)—exposure, probably through preventing tau hyperphosphorylation by regulating p-GSK3 and PP2A level in the cortex and hippocampus (Zhao et al., 2013). Our previous studies have demonstrated that Rg1 and Rb1 intraperitoneal administration to mice for 7 days were both effective in improving memory deficiency induced by scopolamine, and Rg1 was more effective than Rb1 in improving acquisition deficiency in the MWM test. In addition, Rg1 can significantly reduce AChE activity than Rb1, while Rb1 showed stronger effects than Rg1 in adjusting 5-HT level in hippocampus in mice (Wang et al., 2010). These results demonstrated that Rb1 and Rg1 have different neuroprotective mechanisms. So in this study, we assessed the cognitive enhancement effects of Rg1 and Rb1 in SAMP8 (senescence accelerated mice P8) mice to make better understand of their differences.

## Materials and Methods

### Animals

Sixty-six male SAMP8 mice (4-month-old) and 12 male SAMR1 mice (4-month-old) were purchased from the Department of Experimental Animal Science, Peking University Health Science Center (Qualified No. SCXK 2016-0010, Beijing, China). All animals were kept under a controlled environment of 12-h light/dark (8:30 AM–8:30 PM) cycle at 23–25°C and a relative humidity of 50% ± 10%. They had free access to food and water throughout the experiment. All experimental procedures were performed under the approval and supervision of the Care and Use of Laboratory Animals of IMPLAD, CAMS & PUMC, China (SLXD-20181225051), and in accordance with the National Institute of Health Guide for the Care and Use of Laboratory Animals. And all efforts were made to minimize the suffering of the animals.

### Chemicals and Reagents

Ginsenoside Rg1 and ginsenoside Rb1 (purity > 98%) were purchased from Ruifensi Biological Technology Co., Ltd. (Chengdu, China). Donepezil hydrochloride was purchased from Eisai pharmaceutical Co., Ltd (Shanghai, China).

### Experimental Design and Treatment

**Figure 1:**
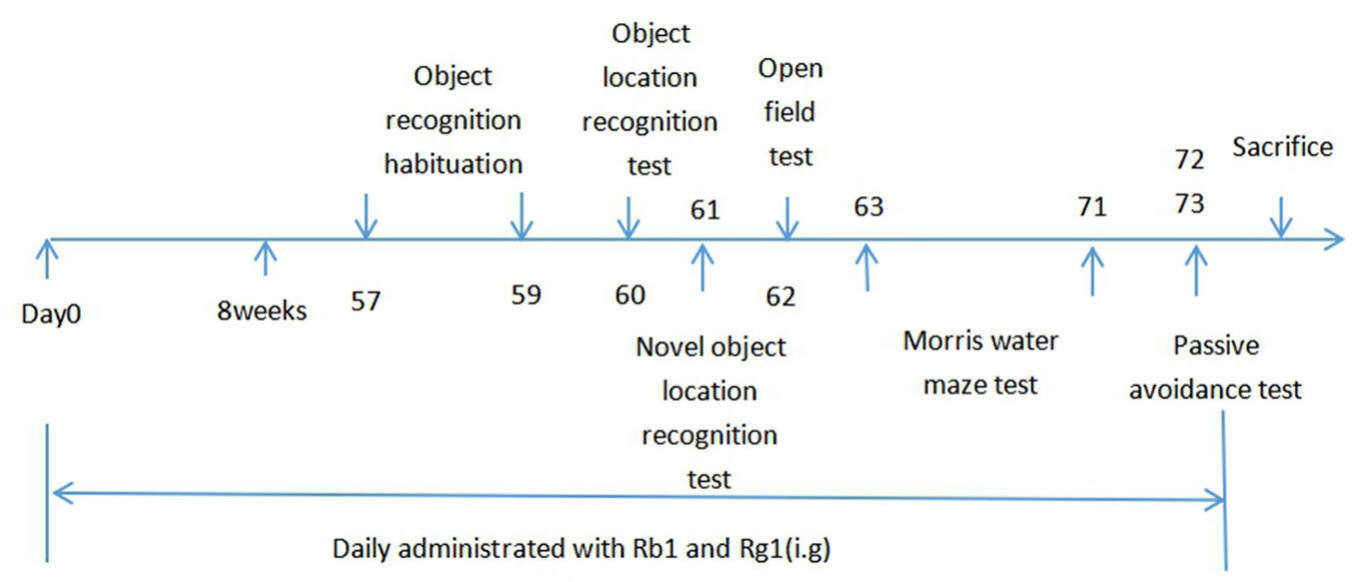
Timeline for the experimental procedure.

One month after breeding, SAMP8 mice were randomly assigned to six groups as introduced in the abstract. The mice in SAMP8 + Rb1, Rg1, or donepezil group were treated daily correspongding drugs for 8 consecutive weeks before behavioral tests. The mice in SAMR1 control group and the SAMP8 model group received orally purified water during the experiment. Rb1, Rg1, and donepezil were prepared with purified water and were administered orally to mice at 0.1 ml/10g per day (**Figure 1**).

All behavioral experiments were conducted after 1 h of drug administration.

### Behavioral Experiments

#### Object Location Recognition (OLR) Test

The OLR test was used to evaluate the short-term and spatial object recognition memory of mice (Ennaceur et al., 2009). The procedure was divided into three stages: habituation, familiarization, and test phases. In the habituation phase, mice were allowed to explore the box (with no objects) for 10 min to acclimatize to the apparatus and reduce the animals' fear of a new environment. The habituation phase was repeated for three consecutive days. On the fourth day, the familiarization and test phases bagan. Firstly, two identical objects were fixed at the adjacent magnets in the chamber, and mice were allowed to freely explore the box for 5 min. Then, after an interval of 30 min, mice were returned to the box in which one of the original objects changed location (“novel”) and the other object remained in the original position (“familiar”), and mice were allowed to freely explore the box for 5 min again. Recognition memory was evaluated by the following formula: DI = (TN - TF)/(TN + TF). DI (discrimination index) refers to the difference between the time exploring the novel and the familiar object, corrected for total time exploring both objects. TN refers to the time spent exploring the “novel” object, and TF refers to the time spent exploring the “familiar” object (Lu et al., 2018a).

#### Novel Object Recognition (NOR) Test

Twenty-four hours after the OLR test, the NOR test was carried out, including the familiarization phase and the test phase, with an interval of 30 min between two phases. The familiarization phase performed similarly to the OLR test. In the test phase, mice were returned to the box in which one of the original objects was replaced by a new one (“novel”) and the other object remained (“familiar”), and mice were allowed to freely explore the box for 5 min. Recognition memory was evaluated by the following formula: DI = (TN − TF)/(TN + TF) corresponding to the difference between the time exploring the novel and the familiar object, corrected for total time exploring both objects (TN = time spent exploring the “novel” object; TF= time spent exploring the “familiar” object) (Lu et al., 2018b).

#### Open Field Test

The open field test used to assess the effects of Rb1 and Rg1 on the locomotor activity for eliminating its interference in cognitive function. The apparatus contain four identical boxes (30 cm × 28 cm × 35 cm). After 30 min of drug administration, each mouse was placed into the center of the box and allowed to explore freely for 3 min. We recorded the total distances and average speeding of the mice in 10 min. The floor of the box was cleaned with 70% ethanol after each trial.

#### Morris Water Maze (MWM) Test

The water maze was consisted of a circular, black pool measuring 100 cm in diameter and 38 cm in height, filled with opaque water (black ink) at the temperature of 23 ± 1°C, with a depth of 25 cm. A submerged (1.5 cm beneath water surface) platform was fixed in the pool (the target quadrant). Objects and staff were fixed as the spatial reference to the mice during the experiment (Yang et al., 2018). The MWM test composed of three sections including the acquisition, the probe trail, and the reverse memory tests.

##### Acquisition Phase (Learning)

In the acquisition test, mice were trained three times daily during 6 consecutive days. Before each train, mice were placed on the hidden platform for 15 s to remember the location of platform. Then, they were put in the pool and allowed to find the platform for 90 s. If mice succeed to find the platform and stay on it for 2 s, it was considered as a successful platform search. If the mice failed to locate the hidden platform within 90 s, the mice were gently guided to the platform, and allowed to stay there for 15 s. The escape latency, the swimming distance, and average speed were recorded every day during the test.

##### Probe Trial (Memory Consolidation)

Twenty-four hours after the acquisition test, the probe trail was conducted to assess spatial reference memory of the animals, in which the platform was removed. The mice were released from the opposite quadrant of target quadrant. The numbers of target crossing of mice within 90 s were recorded to reflect the retention memory for the platform location.

##### Reverse Test (Reverse Memory)

In this section, the platform was moved to the opposite side of the target quadrant to assess the animals' reverse memory (Jinfeng et al., 2013). The process of this test was similar to the acquisition test. The mice were allowed to find the hidden platform in 90 s, and the escape latency to find the platform was recorded. The test was last for 2 days.

#### Passive Avoidance Test

The step-through passive avoidance test box includes a dark chamber and a light chamber with stainless steel grids. The mice encountered an acquisition trail and a consolidation trail. In the acquisition trial, after 3 min habitation, each mouse was put into the test box from the light chamber and allowed to explore 5 min in the test box. When the mouse entered the dark chamber, it will suffer to 0.5 mA electric foot shock in dark chamber. Twenty-four hours later, the consolidation trial was performed as before. The latency and the error times to enter the dark chamber were recorded in mice. This passive avoidance test was used to assess the non-spatial memory in mice.

#### Brain Sample Preparation

After all behavioral tests, mice were anesthetized and sacrificed. The cortical and hippocampal regions were collected and deep frozen in liquid nitrogen followed by transfer to −80°C for further analysis.

#### Brain Immunohistochemistry

Three mice of each group were anesthetized with 5% chloral hydrate and transcardially perfused with 0.9% saline followed by 4% paraformaldehyde. Brains were removed and post-fixed in the same fixative solution for 24 h at 4°C. All immunohistochemistry procedures were conducted according to the previously studies (Aragão et al., 2018; Lamarão-Vieira et al., 2019). Paraffin-embedded sections were dewaxed, hydrated, and rinsed. Then, antigen recovery was performed with citrate buffer solution (pH 6.0), previously heated to 60°C for 23 min. Sections were kept at room temperature for 20 min to decrease the temperature and incubated in 3% hydrogen peroxide solution (H_2_O_2_) for 25 min to block endogenous peroxidase and then incubated with antibodies. After that, sections were incubated with 3% bovine serum albumin (BSA, Solarbio) for 30 min. Primary antibodies were against Iba-1 (Abcam, Ab178847) and GFAP (CST, 12389s). Sections were rinsed three times (5 min/time) with 0.1 M phosphate-buffered saline (PBS, PH 7.4) and revealed with 3, 3-diaminobenzidine (DAB). After DAB staining, sections were counterstained by hematoxylin for 3 min, dehydrate through alcohol gradient and xylene. The sections observed under microscope and the activated cells counted from three different areas in each section.

### Statistical Analysis

All data were analyzed using the SPSS 21.0 software package (IBM, USA) and represented as means ± standard error of the mean (SEM). The data of the acquisition trials in MWM were analyzed using the two-way repeated measures ANOVA, and other data were analyzed by one-way ANOVA followed by multiple *post hoc* comparisons using the least significant difference (LSD) test. P value 0.05 was considered statistically significant difference.

## Results

### Effects of Ginsenosides Rb1 and Rg1 on the OLR Task in SAMP8 Mice

**Figure 2:**
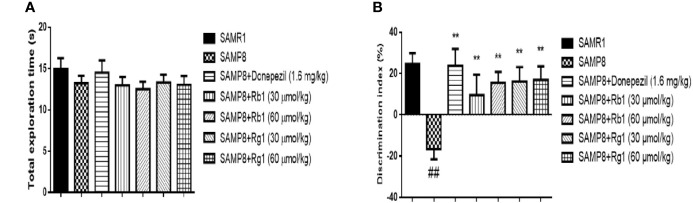
Effects of ginsenosides Rb1 and Rg1 on short-term, spatial memory in object location recognition task. **(A)** Total exploration time in familiarization phase; **(B)** Discrimination index in test phase. All data were expressed as means ± SEM (n = 10–12). ^##^*p* < 0.01versus the SAMR1 group; **p < 0.01versus the SAMP8 group.

In the familiarization phase, **Figure 2A** showed that compared to the SAMR1 mice, SAMP8 mice did not significant differences in the total exploration time (*p* > 0.05). Treatment with Rb1 (30, 60 μmol/kg), Rg1 (30, 60 μmol/kg) and donepezil did not showed significant changes in the total exploration time, which suggested that there were no differences on the ability of exploration and preference for location in mice. In the test phase, the DI of SAMP8 mice significantly decreased (32.79%) compared with the SAMR1 mice, whereas all treatment groups elevated the DI significantly as compared to the SAMP8 group (*p* < 0.01, **Figure 2B**), indicating both Rb1 and Rg1 administrations could improve the object location memory deficit in SAMP8 mice.

### Effects of Ginsenosides Rb1 and Rg1 on the NOR Task in SAMP8 Mice

**Figure 3:**
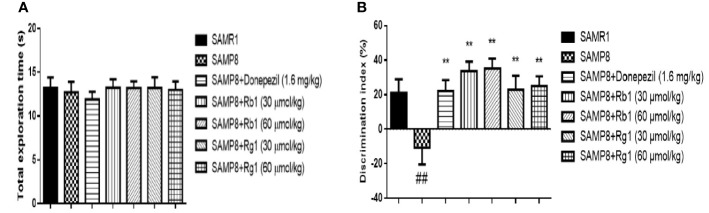
Effects of ginsenosides Rb1 and Rg1 on short‐term, non-spatial memory in novel object recognition task. **(A)** Total exploration time in familiarization phase; **(B)** Discrimination index in test phase. All data were expressed as means ± SEM (n = 10–11). ^##^*p* < 0.01 versus the SAMR1 group; **p < 0.01 versus the SAMP8 group.

As shown in **Figure 3A**, in the familiarization phase, mice in the SAMR1, the SAMP8, the donepezil, the Rg1 and Rb1 treatment groups displayed non-significant similar preference toward two similar objects (*p* > 0.05). These results demonstrated that there was no significant difference on the ability of exploration and preference for object location in mice.

As indicated in **Figure 3B**, the DI of SAMP8 mice was 49.28% significantly lower than those in the SAMR1 group (*p* < 0.01). However, all treatment groups could significantly increase the DI in SAMP8 mice (*p* < 0.01). It illustrated that the SAMP8 mice treated with Rb1 or Rg1 could reverse the NOR memory deficit.

### Effects of Ginsenosides Rb1 and Rg1 on Locomotor Activity of SAMP8 Mice

**Figure 4:**
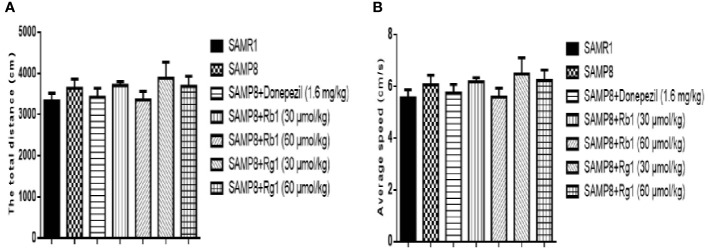
Effects of ginsenosides Rb1 and Rg1 on the locomotor activities in SAMP8 mice. **(A)** The total distance. **(B)** Average speed. All data were expressed as means ± SEM (n = 8–12).

As shown in **Figure 4**, there was no significant difference between the SAMR1 and SAMP8 mice in the total distance (*p* > 0.05) and the average speed (*p* > 0.05). Treatment with Rb1, Rg1, or donepezil did not show significant difference in locomotor activity (*p* > 0.05). Results indicated that cognitive dysfunction did not affect locomotor activity in SAMP8 mice.

### Effects of Ginsenosides Rb1 and Rg1 on Spatial Learning and Memory Deficits in the MWM Test in SAMP8 Mice

**Figure 5:**
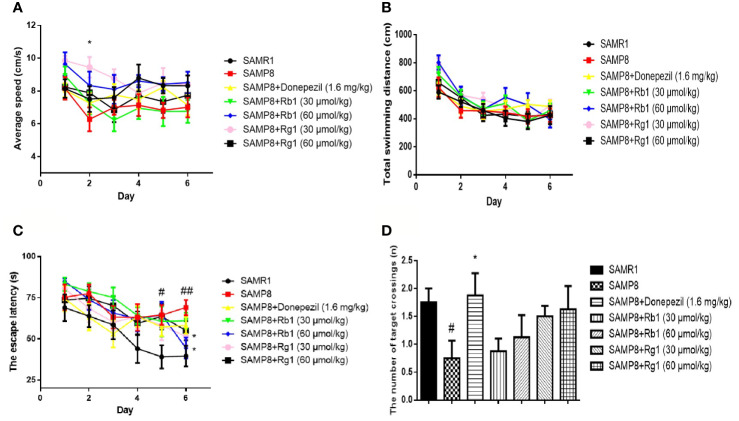
Effects of ginsenosides Rb1 and Rg1 on the spatial learning and memory in the MWM test in SAMP8 mice. **(A)** Average speed. **(B)** Total swimming distance. **(C)** The escape latency. **(D)** The number of target crossings. All data were expressed as means ± SEM (n = 8–11). ^#^*p* < 0.05, ^##^*p* < 0.01versus the SAMR1 group; *p < 0.05 versus the SAMP8 group.

In the acquisition test, as shown in **Figures 5A, B**, there was no significant difference in the average speed and total swimming distance in the SAMR1, SAMP8, donepezil, Rg1 and Rb1 groups from day 1 to day 6, except the average speed of the Rg1 30 μmol/kg treatment group was higher than that of SAMP8 mice on day 2. In **Figure 5C**, the escape latency in the acquisition test in SAMP8 mice was significantly longer than that of the SAMR1 mice from day 5 (44.65%) to day 6 (41.20%), illustrating spatial reference memory impairment in SAMP8 mice (*p* < 0.05, *p* < 0.01). Rb1 (60 μmol/kg) and Rg1 (30 μmol/kg) treatment could signiﬁcantly shorten the escape latency on day 6 (*p* < 0.05). In the probe trial (**Figure 5D**), the number of target crossings of SAMP8 mice was 42.85% lower than the SAMR1 mice (*p* < 0.05), while Rb1 or Rg1 treatment did not show significantly effect in SAMP8 mice.

**Figure 6:**
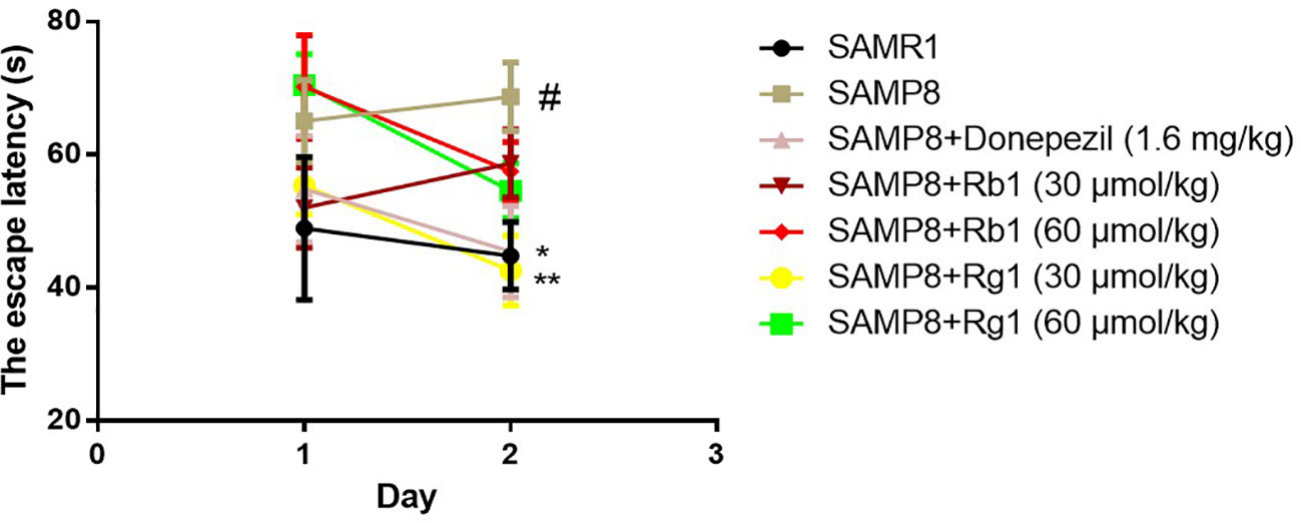
Effects of ginsenosides Rb1 and Rg1 on the relearn ability of SAMP8 mice in the reverse test in MWM test. All data were expressed as the means ± SEM (n = 8–11). ^#^*p* < 0.05 versus the SAM1 group; *p < 0.05, **p < 0.01 versus the SAMP8 group.

As shown in **Figure 6**, the escape latency of SAMP8 mice was 45.79% longer than that of the SAMR1 mice on day 2, illustrating the ability of SAMP8 mice to relearn was affected (*p* < 0.05). Rg1 (30 μmol/kg) or donepezil treatment effectively decreased the escape latency on day 2. These indicated that only Rg1 (30 μmol/kg) could significantly improve the relearn ability dysfunction in SAMP8 mice.

### Effects of Ginsenosides Rb1 and Rg1 on Non-Spatial Memory Deficits in the Passive Avoidance Test

**Figure 7:**
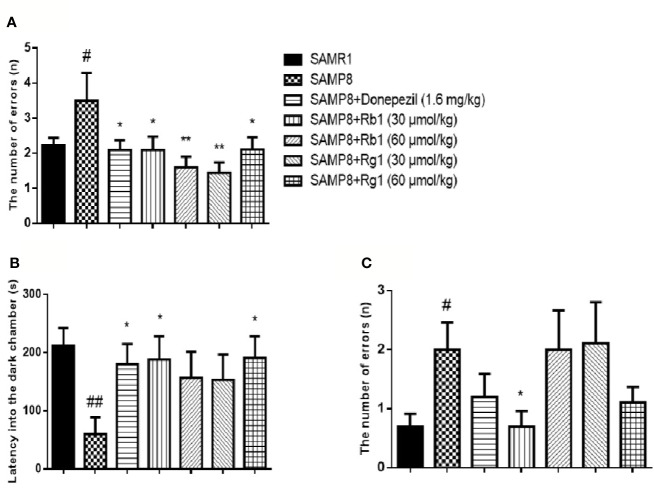
Effects of ginsenosides Rb1 and Rg1 on non-spatial memory deficits in the passive avoidance test in SAMP8 mice. **(A)** The number of errors; **(B)** The latency into the dark chamber; **(C)** The number of errors. All data were expressed as the means ± SEM (n = 8–10). ^#^*p* < 0.05, ^##^*p* < 0.01 versus the SAMR1 group; *p < 0.05, **p < 0.01 versus the SAMP8 group.

In the acquisition phase, as shown in **Figure 7A**, the number of errors in SAMP8 mice was 57.52% higher than the SAMR1 mice (*p* < 0.05). The donepezil, Rb1 (30, 60 μmol/kg), or Rg1 (30, 60 μmol/kg) treatment could significantly reduce the number of errors in SAMP8 mice (*p* < 0.05, *p* < 0.01).

In the test phase (**Figure 7B**), the latency into the dark chamber of SAMP8 mice was 71.57% shorter than the SAMR1 mice (*p* < 0.01), while the donepezil, Rb1 (30 μmol/kg), or Rg1 (60 μmol/kg) treatment effectively extended the latency into the dark chamber in SAMP8 mice. Furthermore, the number of errors in SAMP8 group was more than twice of the SAMR1 mice (*p* < 0.05, **Figure 7C**) and Rb1 (30 μmol/kg) treatment significantly decreased the number of errors in SAMP8 mice (*p* < 0.05).

### Effects of Ginsenosides Rb1 and Rg1 on TNF-α Levels in the Serum and the Cerebral Cortex of SAMP8 Mice

**Figure 8:**
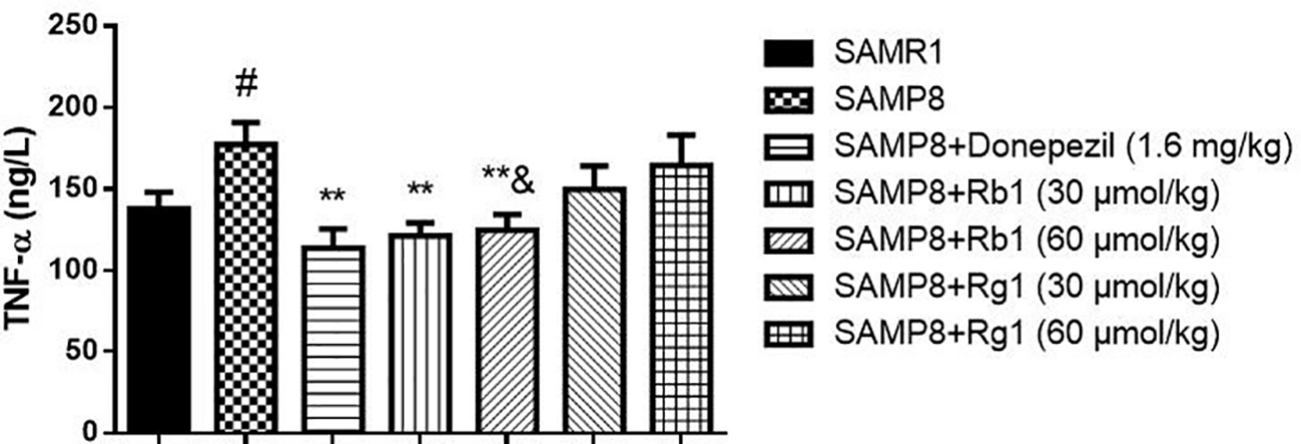
Effects of ginsenosides Rb1 and Rg1 on the TNF-α concentration in the serum of SAMP8 mice. All data were expressed as means ± SEM (n = 6–8). ^#^*p* < 0.05 versus the SAMR1 group; **p < 0.01 versus the SAMP8 group; ^&^*p* < 0.05 versus the Rg1 (60 µmol/kg) group.

As shown in **Figure 8**, the level of TNF-α in the serum of SAMP8 mice was 31.22% higher than of the SAMR1 mice (*p* < 0.05). Treatment with Rb1 (30, 60 μmol/kg) or donepezil significantly decreased TNF-α concentration compared to the no drug treatment SAMP8 mice (*p* < 0.01). However, treatments with Rg1 (30, 60 μmol/kg) had no significant effects on TNF-α production in SAMP8 mice, in spite of a tendency to a lower accumulation of this cytokine. In addition, results showed TNF-α concentration of Rb1 (60 μmol/kg) treatment group was lower than that of Rg1 (60 μmol/kg) treatment group (*p* < 0.05).

**Figure 9:**
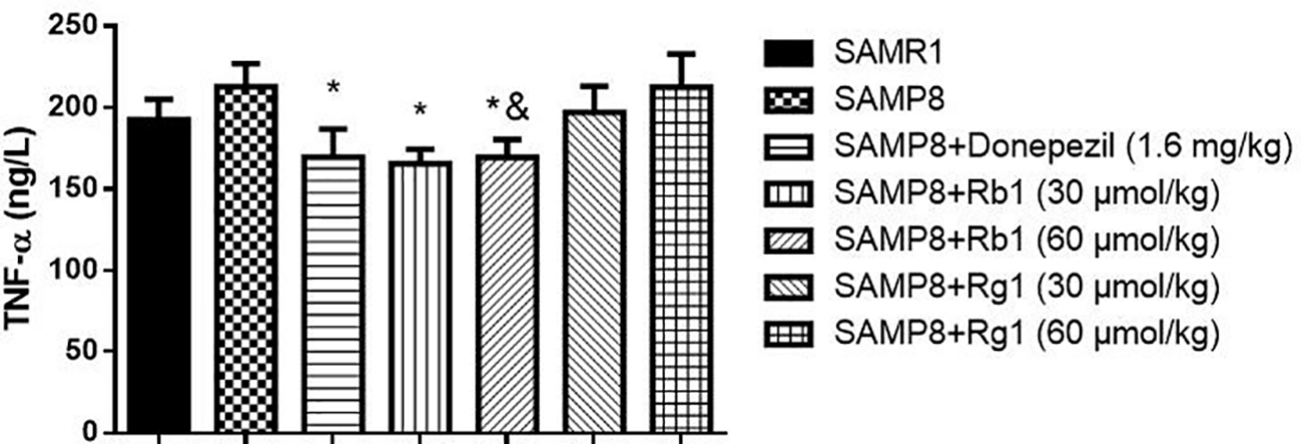
Effects of ginsenosides Rb1 and Rg1 on the TNF-α concentration in the cerebral cortex of SAMP8 mice. All data were expressed as means ± SEM (n = 6–8). *p < 0.05 versus the SAMP8 group; ^&^*p* < 0.05 versus the Rg1 (60 µmol/kg) group.

In **Figure 9**, TNF-α concentration were significantly decreased in the Rb1 (30, 60 μmol/kg) or donepezil treatment groups as compared to the SAMP8 group in the cerebral cortex of mice (*p* < 0.05). The SAMP8 group showed a tendency of higher level of TNF-α than that of the SAMR1 group, but no significant difference.

### Effects of Ginsenosides Rb1 and Rg1 on Avoiding Neuronal Cells Loss in SAMP8 Mice

**Figure 10:**
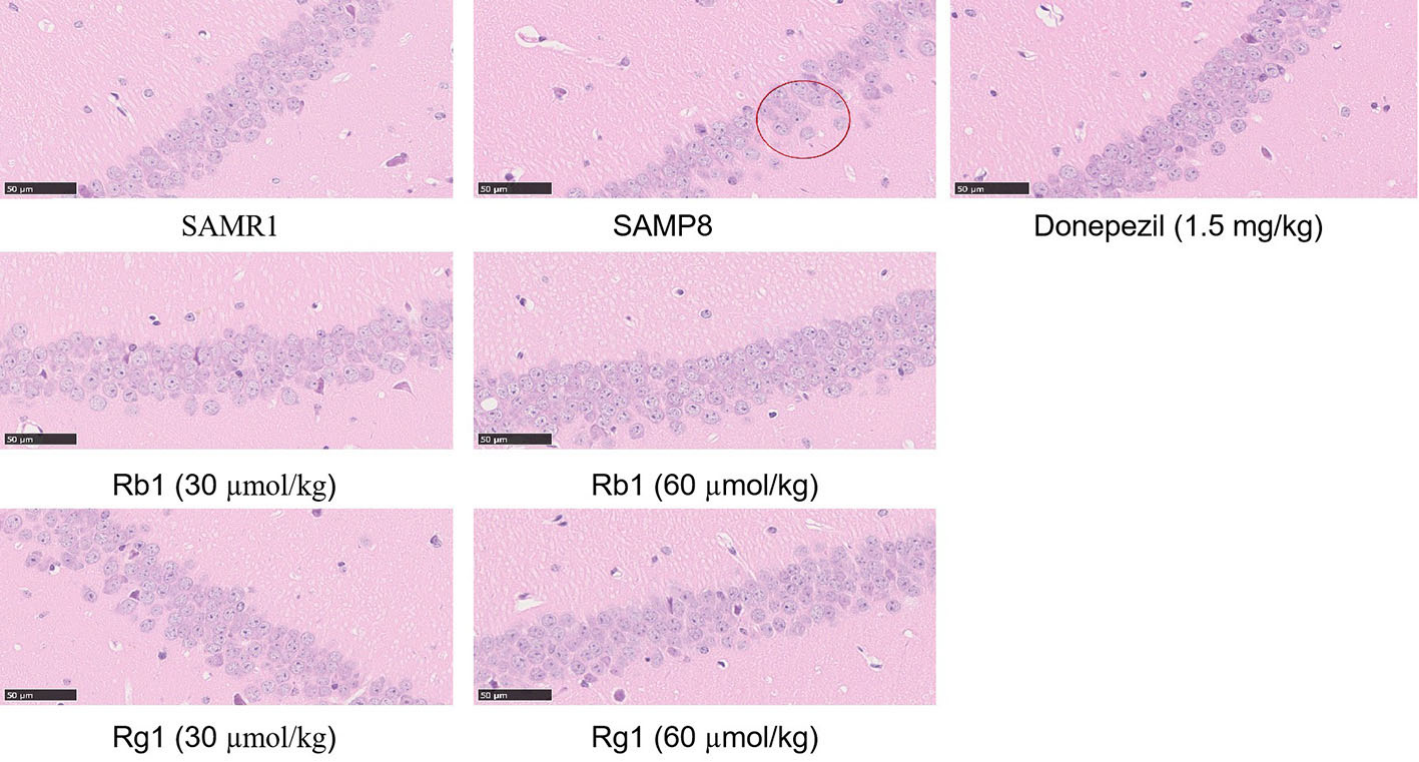
Hematoxylin-eosin staining in the CA1 areas in the hippocampus of SAMP8 mice (×400). All data were expressed as means ± SEM (n = 3).

Representative HE staining results in CA1 areas of the hippocampus are shown in **Figure 10**. In SAMR1 mice, neuronal cells were tightly packed and had large nuclei with clear nucleoli in the hippocampus. In contrast, in SAMP8 group, there were marked morphological changes: neurons were loosely organized and uneven cytoplasm distribution in the hippocampus. In Rg1 or Rb1 treatment group, neuronal cells had large nuclei, and arranged in an orderly manner, indicating that ginsenoside Rg1 or Rb1 treatment could reduce the loss of neuronal cells.

### Immunohistochemistry Studies

#### Analysis of Microglias Activation

**Figure 11:**
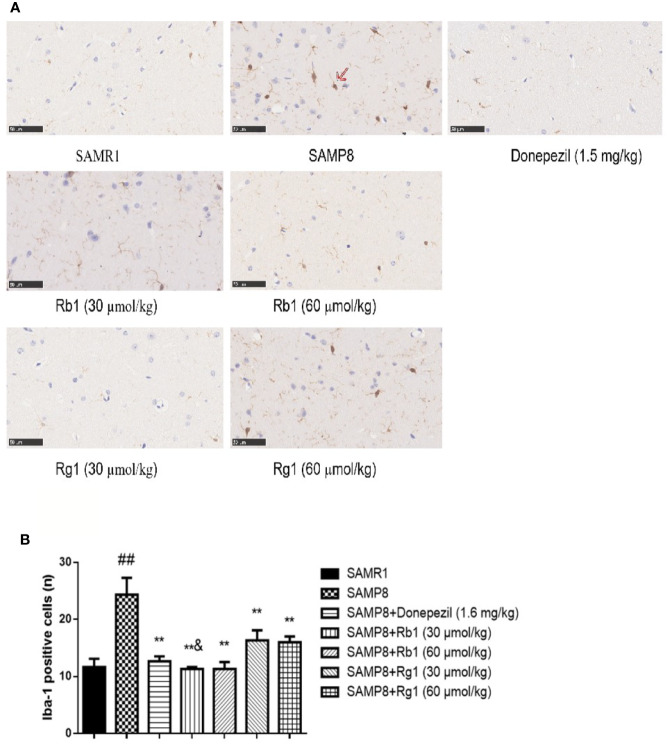
Effects of ginsenosides Rb1 and Rg1 on microglias in the hippocampus of SAMP8 mice. **(A)** Immunohistochemical staining of Iba-1 in the hippocampus of mice brain (×400). **(B)** The number of Iba-1 positive cells. All data were expressed as means ± SEM (n = 3). ^##^*p* < 0.01 versus the SAMR1 group; **p < 0.01 versus the SAMP8 group; ^&^*p* < 0.05 versus the Rg1 (30 µmol/kg) group.

As shown in **Figure 11A**, the number of activated microglia cells in the hippocampus of the SAMP8 mice was more than twice of the SAMR1 mice (*p* < 0.01). Microglial cells were brown after being labeled by Iba-1, and activated cells were bigger and intensively stained. Donepezil, Rb1, or Rg1 treatment significantly decreased the number of activated microglia cells in SAMP8 mice (**Figure 11B**). Compared with the Rb1 treatment, Rg1 treatment showed lower effect on the reduction of the activation of microglia cells in SAMP8 mice.

#### Analysis of Astrocytes Activation

**Figure 12:**
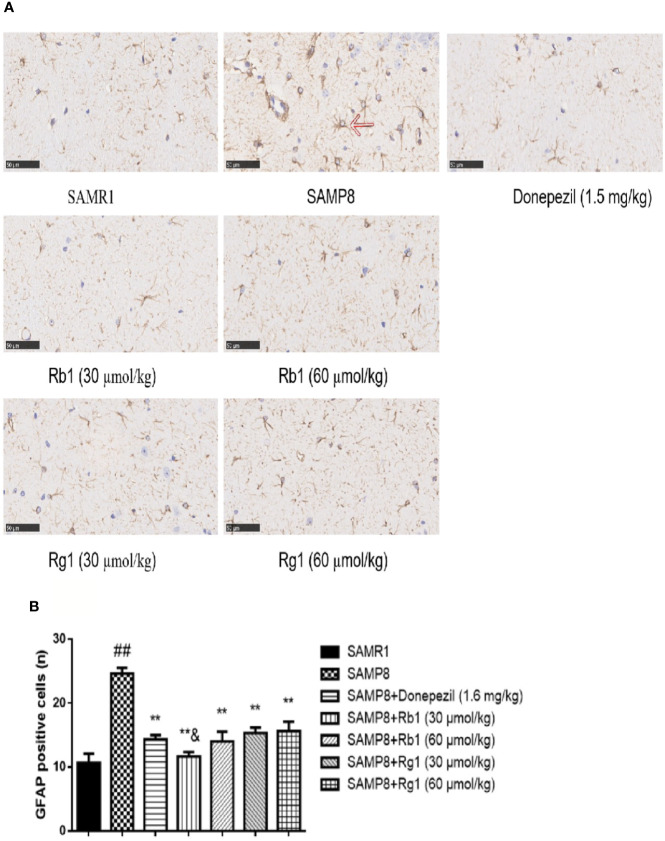
Effects of ginsenosides Rb1 and Rg1 on astrocytes in the hippocampus of SAMP8 mice. **(A)** Immunohistochemical staining of GFAP in the hippocampus of mice brain (×400). **(B)** The number of GFAP positive cells. All data were expressed as means ± SEM (n = 3). ^##^*p* < 0.01 versus the SAMR1 group; **p < 0.01 versus the SAMP8 group; ^&^*p* < 0.05 versus the Rg1 (30 µmol/kg) group.

Compared with the SAMR1 mice, the activated astrocytes in the hippocampus of the SAMP8 mice were more than doubled (*p* < 0.01, **Figure 12**). GFAP positive expression cells proliferated significantly, and these cells had larger nuclei and shorter protrusions, and were darkly stained. In the SAMR1 mice, astrocytes were mostly in a non-activated state. After treatment with donepezil, Rb1, or Rg1, the number of activated astrocytes in SAMP8 mice dramatically decreased. The number of activated astrocytes in the Rb1 (30 μmol/kg) treatment group was significantly lower than that of the Rg1 (30 μmol/kg) treatment group (*p* < 0.05).

### Effects of Ginsenosides Rb1 and Rg1 on Inhibiting NLRP3 Inflammasome Assembly

**Figure 13:**
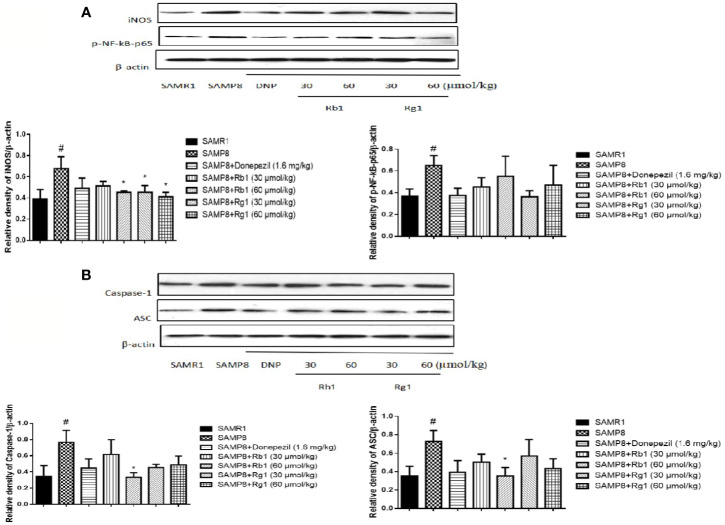
Effects of ginsenosides Rb1 and Rg1 on the protein levels of p-NF-kB-p65, ASC, caspase-1, iNOS in the hippocampus of SAMP8 mice. **(A)** Protein expression and gray intensity analysis of p-NF-kB-p65 and iNOS; **(B)** Protein expression and gray intensity analysis of ASC and caspase-1. All data were expressed as means ± SEM (n = 3). ^#^*p* < 0.05 versus the SAMR1 group; *p < 0.05 versus the SAMP8 group.

NF-kB-mediated cell signaling is one of the common pathways engaged in the inflammatory response. The expression of p-NF-kB-p65 (*p* < 0.05), ASC (*p* <, 0.05), and caspase-1 (*p <* 0.05) in the hippocampus of SAMP8 mice were twice level as that of SAMR1 mice (*p* < 0.05). Rb1 (60 µmol/kg) treatment could decrease the expression of ASC and caspase-1 in the hippocampus of SAMP8 mice (**Figure 13B**, *p* < 0.05).

As shown in **Figure 13A**, the protein expression of iNOS in SAMP8 mice was more than twice level as that of the SAMR1 mice (*p* < 0.05). Treatment with Rb1 (60 µmol/kg) or Rg1 (30, 60 µmol/kg) significant attenuated this increase in SAMP8 mice (*p* < 0.05).

### Effects of Ginsenosides Rb1 and Rg1 on the Accumulation of Aβ in Hippocampus

**Figure 14:**
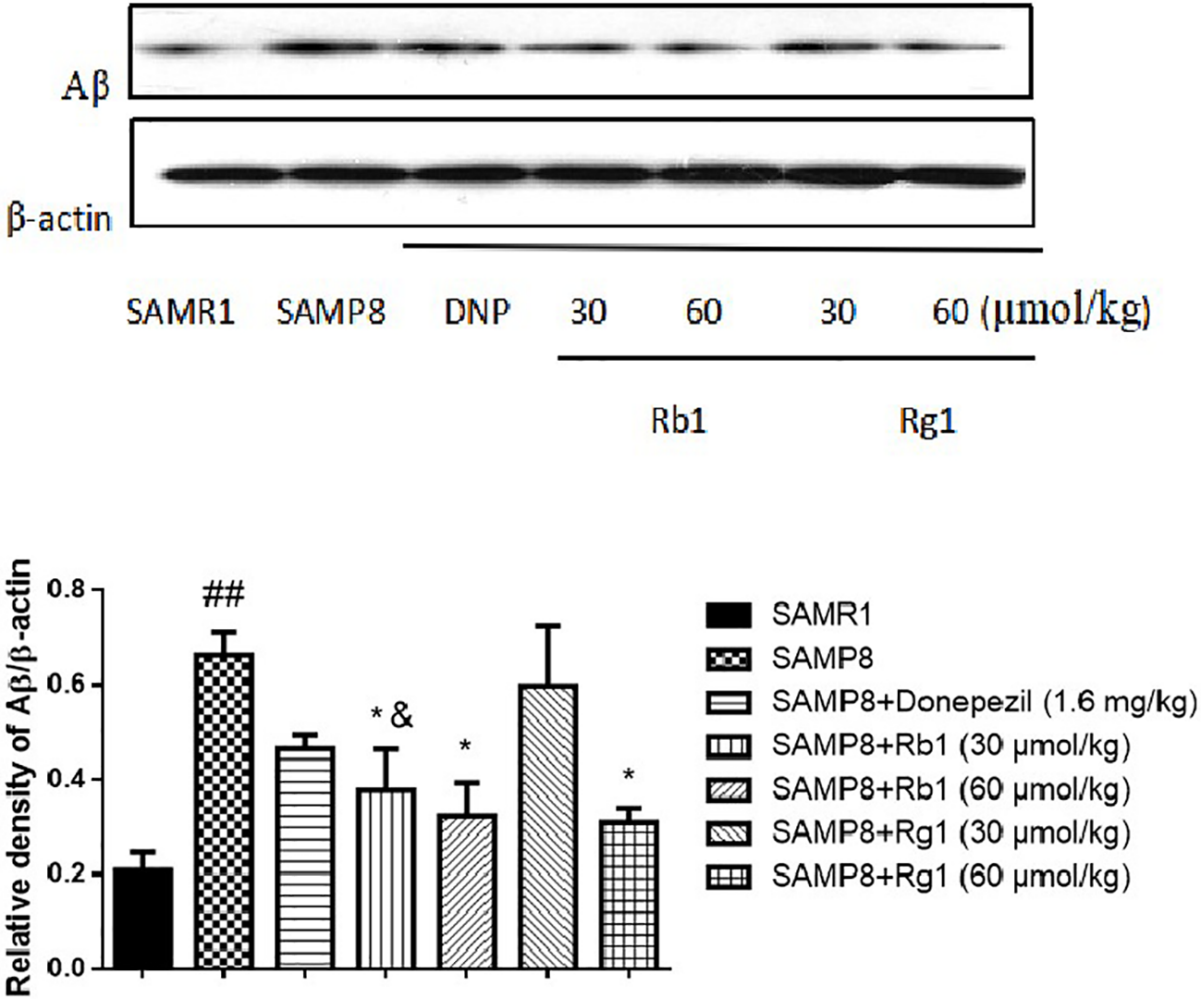
Effects of ginsenosides Rb1 and Rg1 on the protein level of Aβ in the hippocampus of SAMP8 mice. All data were expressed as means ± SEM (n = 3). ^##^*p* < 0.01 versus the SAMR1 group; *p < 0.05 versus the SAMP8 group; ^&^*p* < 0.05 versus the Rg1 (30 µmol/kg) group.

As shown in **Figure 14**, compared with the SAMR1 mice, the protein level of Aβ in the hippocampus of SAMP8 mice was significantly increased three times higher than the former (*p* < 0.01). Rb1 (30, 60 µmol/kg) or Rg1 (60 µmol/kg) treatment significantly reduced the expression of Aβ in SAMP8 mice (*p* < 0.01), indicating Rb1 and the high dose of Rg1 could significantly reduce the accumulation of Aβ in SAMP8 mice. Besides, Rb1(30 µmol/kg) has a more prominent inhibitory effect on reducing the protein level of Aβ compared to the same dose of Rg1 (*p* < 0.05).

## Discussion

Our study showed that ginsenosides Rb1 and Rg1 could ameliorate memory impairments in SAMP8 mice and suggested that their action may be mediated *via* modulation of NLRP3 inflammasome, decreasing TNF-α level, astrocytes, and microglias activation, ameliorating oxidative stress, and inhibiting expression of p-NF-kB-p65, ASC, caspase-1, and iNOS in hippocampus. Besides, amyloid β protein (Aβ), a major component of the senile plaque, is central to the pathogenesis of AD. Treatments with Rb1 and Rg1 significantly reduced the production of Aβ in the brain of SAMP8 mice. In addition, our results showed for the first time that Rb1 has stronger anti-inflammatory effects than Rg1 in SAMP8 mice.

The SAMP8 is a classic accelerated aging model mouse (Gao et al., 2019). It shows significant age-related impairment in memory and learning ability (Akiguchi et al., 2017), and demonstrated many pathological features similar to those of AD patients such as altered amyloid β (Aβ) proteins (Morley et al., 2002), elevated phosphorylation of tau (Canudas et al., 2005), as well as synaptic and dendritic pathological changes (Nomura, 1999). In addition, SAMP8 mice show severe inflammatory reactions and oxidative stress related to aging, which can exacerbate neurological damage and cognitive decline (Jiang et al., 2018; Puigoriol-Illamola et al., 2018). Moreover, compared with same age of SAMR1 mice, SAMP8 mice show significant emotional and memory impairments after six months of age (Shih-Yi et al., 2018). Previous studies exhibited that this strain of mice accelerated aging from 4 months, showing learning and memory disorders, and being in a low-stress and low-terror dementia state with aging (Wang Miyamoto, 1997; Feng et al., 2009). In our study, we found SAMP8 mice showed age-associated behavioral impairments at 6 months, such as short-term, long-term, spatial and non-spatial learning, and memory deficits. These results are consistent with previous studies (Miyamoto et al., 1986; Hosokawa et al., 1997). Furthermore, SAMP8 mice at 6 months of age showed poor performance in ORT test (Adler et al., 2014; Jin et al., 2016), and our treatment of Rb1 and Rg1 effectively enhanced DI in both OLR and NOR tests, which coincide with previous study on Rb1 and Rg1, indicating their positvie effects on promote learning and memory in normal animals or animals with cognitive impairment (Qiong et al., 2014). However, our study for the first time demonstrated that both Rb1 and Rg1 could significantly ameliorate impaired short-term memory in SAMP8 mice, and the two ginsenosides have similar improvement effects.

MWM test was a classical behavioral method for evaluating the reference, spatial, and working memory of rodents (Meiri and Rosenblu, 1998; Yongliang et al., 2018; Ahmed et al., 2019). It was reported that SAMP8 mice showed spatial memory impairment from 6 months of age (Qin et al., 2008). The results of the acquisition test suggest that the escape latency in the SAMP8 group was significantly longer than the SAMR1 group from day 5 to day 6, and the number of target crossing decreased in the probe trial. These results indicated that 6-month-old SAMP8 mice showed significantly decreased reference memory and long-term spatial memory, compared with the SAMR1 mice. We used the reversal trial to evaluate the reversal learning ability of mice, the reversal learning was significantly damaged in 6-month-old SAMP8 mice, which are in agreement with a previous study (Ding et al., 2019). Many reports have confirmed that both Rb1 and Rg1 have neuroprotective effects and can improve cognitive function in AD animal models (Li et al., 2015; Lulin et al., 2017; Wang et al., 2018). Our study demonstrated that Rb1 treatment significantly ameliorated reference memory impairment in SAMP8 mice in the acquisition trial task by shorting the escape latency, but Rg1 treatment showed effectively shortened the latency after training to find the hidden platform. Zhang Juntian used a variety of methods and models to observe the effects of ginseng on the learning and memory in mice, demonstrating that ginsenoside Rg1 can promote memory acquisition, consolidation and reproduction, while Rb1 mainly improves memory acquisition and reproduction (Zhang, 2000). This study showed that after treatments with Rb1 and Rg1 for 8 weeks, Rb1 has a stronger effect than Rg1 on improving the reference memory in SAMP8 mice. Although both Rb1 and Rg1 didn't significantly ameliorate the spatial memory in the probe trial, in the reverse memory test, we found Rg1 could significantly decrease the latency to reach a hidden platform in SAMP8 mice.

The non-spatial memory improvement effect of Rg1 was reported in previous researches, which found Rg1 improved scopolamine (SCOP) induced memory impairment in a step-through test (Yu-Zhu Wang et al., 2009) and both Rg1 and Rb1 can improve the impaired memory performance induced by SCOP in step-down passive avoidance test (Wang et al., 2010). From the age of four months, SAMP8 mice showed the existence of learning deficits in passive avoidance test (Lourdes et al., 2014; Ren et al., 2018). In this study, the latency enter to the dark chamber of SAMP8 mice was significantly shorter than the SAMR1 mice, which showed for the first time that Rb1 has stronger effects than Rg1 on improving non-spatial memory deficits in SAMP8 mice in the passive avoidance test.

Growing body of evidence indicates that neuroinflammation plays a key role in the prevalence and severity of AD, and its role in the pathogenesis of AD is even greater than that of senile plaques and nerve fiber entanglements (Heneka et al., 2015). Astrocytes and microglias are the main non-neuronal cells that mediate neuroinflammation (Stephenson et al., 2018). Microglias are belong to the mononuclear phagocyte family and widely considered to be the main immune effectors in the central nervous system (Giulian, 1987). Astrocytes are key regulators of the brain's inflammatory response and reactive astrogliosis is a universally acknowledged feature of AD (Frost and Li, 2017). Activation of astrocytes and microglias result in the production of major proinflammatory cytokines (TNF-α, IL-6, or IL-1β) and neurotoxic factors (reactive oxidative species and tumor necrosis factor-α), which are typically associated with neurodegenerative diseases including AD (Lull and Block, 2010; Calabrese et al., 2011a). Nuclear factor-κB (NF-κB) is a family of transcription factors that have a crucial role in inflammation, survival, and apoptosis. NF-κB has been shown to be activate by transcribing the genes encoding pro-inflammatory cytokines, inducible nitric oxide synthase (iNOS), cyclo-oxygenase-1, and cyclo-oxygenase-2. All these induced a consequent increase in inflammation-mediated signals (Calabrese et al., 2011b; Scuto et al., 2019). A few studies have found that oxidative stress (Baltanás et al., 2013), high levels of inflammation (Ren et al., 2018), activated microglias and astrocytes (Chen et al., 2018) were found in brain tissue of SAMP8 mice, especially the hippocampus. Rb1 and Rg1 were the active ingredients of the root of Panax ginseng C. A. Mayer, which have anti-inflammatory and anti-oxidative stress pharmacological effects (Hui et al., 2017; Xu et al., 2018). Rg1 can decrease the expression of ASC, caspase−1, caspase−5, IL−1β in the hippocampus in mice injury induced by chronic glucocorticoids exposure (Zhang et al., 2017). Interestingly, our results showed that administration of Rb1 significantly inhibited the production of TNF-α compared with the SAMP8 mice, while Rg1 couldn't. In addition, our data indicated strong anti-inflammatory activity of Rb1 and Rg1 on inhibiting activated microglias and astrocytes in the hippocampus of SAMP8 mice, and Rb1 has more stronger effects than Rg1. Rb1 is different from Rg1 in chemical structure, Rb1 contains four sugars but Rg1 only has two (Wang et al., 2010). Some reports on the neuroprotective activity of these compounds implied their capacity to cross BBB, but Rb1 and Rg1 showed low capacity to permeate towards the brain. In the *in vitro* BBB model, ginsenoside Rb1 showed better permeation than Rg1 (Fernández-Moriano et al., 2017). Rb1 mainly regulates stress and Rg1 mainly improves learning and memory (Zhang, 2000). Therefore, in SAMP8 mice, Rb1 still plays a major role in regulation of stress. Our study compared for the first time the effects of two ginsenosides on the improvement of neuroinflammation in SAMP8 mice.

The inflammasome has been identified as a multi-protein complex which plays a key role in innate immune responses against pathogens and toxic metabolites (Nakanishi et al., 2018). Among those types of inflammasomes, NOD-like receptor pyrin domain containing 3 (NLRP3) possesses a critical role in inflammatory response (Li et al., 2018), and associated with many diseases, including AD, Parkinson's disease, diabetes, atherosclerosis, and cerebral ischemia/reperfusion injuries (Guo et al., 2016; Feng et al., 2018; Li et al., 2019; Nasonov and Popkova, 2018; von Herrmann et al., 2018). NLRP3 activation induces aggregation of ASC, leading to caspase-1 activation, promoting the release of inflammatory factors (Stancu et al., 2019). Furthermore, it was reported that Aβ can directly interacted with NLRP3, leading to the activation of the NLRP3 inflammasome, resulting in the activation of caspase-1, and microglial activation (Kayed et al., 2003; Halle et al., 2008). In this study, we found that the expression of ASC and caspase-1 in the hippocampus of SAMP8 mice were significant increased, these are consistent with the findings of Jing Jiang (Jiang et al., 2018) and Ning Ding (Ning et al., 2017). The iNOS in the central nervous system is pathologic and plays an important role in neuroinflammation (Kim et al., 2018). Increasing iNOS expression was found in cultured astrocytes by Aβ, and it could result in excessive production of NO, causing neuronal damage and death (Wang et al., 2017). In our results, the protein level of iNOS in the hippocampus of Rb1 and Rg1 treatment mice were lower than that of the SAMP8 mice, which suggest Rb1 and Rg1 could inhibit the neuroinflammation in SAMP8 mice. The accumulations of Aβ were found in AD animal models, such as aging mice (Li et al., 2016), transgenic mice with AD (Dionísio et al., 2015), and over-expression of Aβ is often one of the important factors that promote chronic inflammation and oxidative stress in the brain (Batarseh et al., 2016). Our results showed the significant increase of Aβ expression in the hippocampus of SAMP8 mice, indicating the neuroinflammation associated with overexpression of Aβ. We found Rb1 and Rg1 treatment could decrease the overexpression of Aβ. Previous researches demonstrated that Rg1 may involve in the activation of Akt/ERK 1/2 and PKA/CREA signaling pathways, and the signal-regulated NF-kB/NO signaling pathways, enhance neurite outgrowth and protect against Aβ induced damage (Huang et al., 2016; Wu et al., 2016; Hejian et al., 2019). Rb1 could improve cognitive and memory functions by inhibiting the levels of pro-apoptosis mediators and improving the levels of anti-apoptosis mediators in the rat model of AD induced by Aβ 1-40 (Wang et al., 2018). In addition, Rb1 showed a significant effect in reducing caspase-1 and ASC protein expression, indicating Rb1 can regulate the inflammatory reaction in the brain by inhibiting ASC and caspase-1.

In summary, although there are several studies have reported that Rg1 and Rb1 are both effective in improving memory impairment of AD animal models, especially in SAMP8 mice, there was no report regarding the different protective effects of Rg1 and Rb1 in facilitating neuroinflammation of SAMP8 mice at the same dosage level. Our present study demonstrated that: (1) Rb1 and Rg1 have similarly effects on improving the short-term, spatial and non-spatial memory in SAMP8 mice. (2) Rg1 was more effective than Rb1 in improving escape acquisition and reverse memory deficiency in SAMP8 mice. (3) For the first time, we found that Rb1 has stronger effects than Rg1 on improving non-spatial memory in SAMP8 mice. (4) Rb1 showed stronger effects than the same dose of Rg1 in inhibiting glial cell activation, the accumulation of Aβ and NLRP3 inflammasome relative protein in the hippocampus of SAMP8 mice. (5) Rg1 was more effective than Rb1 in inhibiting the expression of iNOS in the brain of SAMP8 mice. These results indicated that Rb1 might be more potent than Rg1 on improving the acquisition impairment and has stronger effect in inhibit neuroinflammation. However, further study needed to do in order to make better explanation the difference of Rg1 and Rb1 in cognitive enhancement.

## Data Availability Statement

All data generated for this study are included in the article.

## Ethics Statement

The animal study was reviewed and approved by the Care and Use of Laboratory Animals of IMPLAD, CAMS & PUMC, China (SLXD-20181225051). The animal study was reviewed and approved by the Care and Use of Laboratory Animals of IMPLAD, CAMS & PUMC, China (SLXD-20181225051).

## Author Contributions

The authors contributed in the following way: YY performed experiments, wrote manuscript and analyzed data; SL drafted the first version of the manuscript; HH and JL offered experimental help; YL analyzed part of the data; SC and AP revised the manuscript; XL and QW designed the study and revised the manuscript. All authors have read and approved the final manuscript.

## Funding

This work was supported by the National Key Research and Development Program of China (2016YFE0131800), Science & Technology department of Sichuan province (2019YFH0023), Office of Sciences & Technology and Talent work of Luzhou (2018LZXNYD-ZK32), the High - end Talents Recruitment Program (Liu Xinmin group) of Luzhou Municipal People's Government.

## Conflict of Interest

The authors declare that the research was conducted in the absence of any commercial or financial relationships that could be construed as a potential conflict of interest.
